# Strength of Word-Specific Neural Memory Traces Assessed Electrophysiologically

**DOI:** 10.1371/journal.pone.0022999

**Published:** 2011-08-10

**Authors:** Alexander A. Alexandrov, Daria O. Boricheva, Friedemann Pulvermüller, Yury Shtyrov

**Affiliations:** 1 Saint Petersburg State University, Saint Petersburg, Russian Federation; 2 Medical Research Council (MRC), Cognition and Brain Sciences Unit, Cambridge, United Kingdom; University of Southern California, United States of America

## Abstract

Memory traces for words are frequently conceptualized neurobiologically as networks of neurons interconnected via reciprocal links developed through associative learning in the process of language acquisition. Neurophysiological reflection of activation of such memory traces has been reported using the mismatch negativity brain potential (MMN), which demonstrates an enhanced response to meaningful words over meaningless items. This enhancement is believed to be generated by the activation of strongly intraconnected long-term memory circuits for words that can be automatically triggered by spoken linguistic input and that are absent for unfamiliar phonological stimuli. This conceptual framework critically predicts different amounts of activation depending on the strength of the word's lexical representation in the brain. The frequent use of words should lead to more strongly connected representations, whereas less frequent items would be associated with more weakly linked circuits. A word with higher frequency of occurrence in the subject's language should therefore lead to a more pronounced lexical MMN response than its low-frequency counterpart. We tested this prediction by comparing the event-related potentials elicited by low- and high-frequency words in a passive oddball paradigm; physical stimulus contrasts were kept identical. We found that, consistent with our prediction, presenting the high-frequency stimulus led to a significantly more pronounced MMN response relative to the low-frequency one, a finding that is highly similar to previously reported MMN enhancement to words over meaningless pseudowords. Furthermore, activation elicited by the higher-frequency word peaked earlier relative to low-frequency one, suggesting more rapid access to frequently used lexical entries. These results lend further support to the above view on word memory traces as strongly connected assemblies of neurons. The speed and magnitude of their activation appears to be linked to the strength of internal connections in a memory circuit, which is in turn determined by the everyday use of language elements.

## Introduction

Language is one of the least understood functions of the human brain. Unlike neural substrates of, for example, somatosensory or motor systems, even the very nature of linguistic representations in the brain remains hotly debated. How are words represented in the human brain, and can these representations be reliably quantified? During the last few decades, substantial progress in this area of research was achieved in the rapidly developing field of cognitive neuroscience. Neurophysiological experiments, especially those using fast imaging tools such as electroencephalography (EEG), which can track neural activity with high temporal resolution, were able to suggest both temporal aspects and structural bases underlying various linguistic processes (for reviews, see e.g. [1], [2]).

In delineating the mechanisms our brain uses to store and access spoken words and morphemes in the mental lexicon [3], a number of recent experiments have used the passive oddball paradigm, in which the subjects are presented with linguistic contrasts between frequent (so-called ‘standard’) and unexpected rare (‘deviant’) stimuli, without any stimulus-related task or stimulus-oriented attention [4]. These acoustic contrasts generate the so-called mismatch negativity (MMN) response, a subcomponent of auditory event-related potential, ERP [5]. The main motivations for applying MMN to exploring the brain foundations of lexical access are [6], [7]: (i) its automaticity (meaning that its contamination/masking by neural correlates of stimulus-driven strategies or attention variation is limited); (ii) its specificity to individual sounds (allowing the researcher to scrutinize response patterns for individual lexical entries), and (iii) the MMN being a response to acoustic contrasts (which allows one to incorporate identical acoustic contrasts into different contexts, thus helping to mitigate stimulus-related acoustic confounds). The downsides of the MMN approach include the need to repeat stimuli to maximize signal-to-noise ratios, and the related difficulty in generalizing findings based on a single-item approach. However, the results of MMN studies could be cross-validated using more conventional paradigms [2], whilst the evidence provided by single item results cannot be denied per se.

A body of studies applying the MMN approach to linguistic materials established it as a valuable tool with which to study the neural correlates of lexical access. When the experimental volunteers were presented with acoustically matched word and pseudoword stimuli, an increased MMN response was found when the deviant stimulus was a meaningful word as opposed to an acoustically matched phonotactically legal pseudoword [8], [9], [10], [11]. This so-called “lexical enhancement” of the MMN, which typically peaks at 100–200 ms, has been demonstrated by different groups using different apparatuses, stimulation sequences, and a variety of languages [12], [13], [14], [15], [16], [17]. Superior- and midde-temporal sources of this lexical enhancement were recently suggested by functional magnetic resonance imaging [18], whilst electro- and magnetoencephalographic studies are more suggestive of a larger fronto-temporal network [19]. More detailed investigations have also shown that the MMN may be sensitive to more than just lexicality and can serve as an index of word-category-specific processing, supporting, for example, the notion of early processing and representational differences between verbs and nouns [20].

This increased response to meaningful words under non-attend conditions was clearly in need of explanation, as it appeared to contradict the well-known phenomenon of a larger MMN for unexpected deviant acoustic stimuli [5]. While it still may be possible to explain some of the linguistic increase in MMN with phonological familiarity [21], [22], those earlier studies that precisely controlled for phonological and psycholinguistic properties linked the word-elicited MMN enhancement to lexico-semantic properties of the stimuli. They suggested that this enhancement is a correlate of the activation of cortical memory traces for words. Such memory traces are conceptualized as distributed, strongly connected populations of neurons. The lexical ERP enhancement was thus interpreted as a neurophysiological signature of long-term memory traces for words in the brain that become automatically activated when the word is presented, even if it is not in the focus of one's attention [6], [7]. Such lexical traces are formed as a consequence of the frequent use of words (in both perception and production), which, through Hebbian associative learning, links participating active neurons into neuronal circuits with strong internal connections [23], [24], [25]. These robust connections can support the circuit activity even under low-attention conditions and provide the neuronal implementation of long-term memory traces. To put it simply, strongly connected neuronal networks that act as memory traces for words or morphemes (i.e., entries in the mental lexicon) generate stronger neurophysiological responses than acoustically similar pseudowords that lack such an underlying representation [26].

This theoretical framework makes a clear prediction that the amount of neural activation elicited by a particular word depends on the strength of its lexical representation in the brain. More frequent use of a word should lead to a more strongly connected neuronal ensemble, whereas a less frequently used item would be associated with a more weakly linked circuit. Words with higher occurrence frequencies in the individual's language should therefore lead to a more pronounced lexical MMN response than rare words. This prediction was tested in the current study: using EEG we compared brain responses to words with low and high standardized lexical frequencies presented as rare deviants in the passive oddball paradigm while keeping physical stimulus contrasts identical. Such an experiment can serve as a more refined test for the above distributed account of word-specific memory traces, which to date has been supported by the cruder word-pseudoword differences found in previous studies. More generally, the effects of word frequency on brain responses elicited by spoken words have been rarely investigated; most previous research has concentrated on frequency effects in the visual modality (e.g. [27]). Even less is known about possible word frequency effects in the auditory modality under conditions of limited attention.

## Materials and Methods

### Subjects

Ten healthy right-handed native Russian speakers participated in the experiments (7 females, age range 19–22, mean 19.3). All volunteers reported normal hearing and no history of neurological disorders or drug abuse. All subjects gave their informed written consent, and the experiments were performed in accordance with the Declaration of Helsinki with approval of the University of St. Petersburg Ethics Committee.

### Stimuli

As experimental stimuli, we used two consonant-vowel-consonant (CVC) words with different frequencies of occurrence in the Russian language. Word frequency was estimated according to the word frequency dictionary of the Russian language [28]. In one condition, a high-frequency word *<$>\scale 85%\raster="rg2"<$>* (*mir*, /m'ir/, English: *world*) with a lemma frequency of 569.14 ipm (instances per million words) and word-form frequency of 200.40 ipm was used as a rare, unexpected, deviant stimulus. A low-frequency word *<$>\scale 85%\raster="rg3"<$>* (*mor*, /mor/, English: *famine*, *plague*) with a lemma frequency of 4.22 ipm and word-form frequency of 1.44 ipm served as the frequent standard stimulus. In the other condition, a reversed design was applied (i.e., the low-frequency word *mor* was presented as the deviant stimulus and the high-frequency word *mir* was the standard). Thus, the standard–deviant acoustic-phonetic contrast, the critical variable determining the MMN response [29], was identical in both conditions, while the MMN responses were elicited either by high- or low-frequency deviant items. As previous research suggests, the lexical status of the deviant stimulus plays a critical role in word-elicited MMN, whereas that of the standard has little or no significance [9]. Every subject was exposed to the two experimental conditions, whose order was counter-balanced across the subject group.

To further validate the stimuli, we also estimated diphone and triphone frequencies for the phoneme combinations within the two words. Whereas occurrences of the diphones, /m'i/ and /mo/, were similarly high in frequency (14890 vs. 17860 ipm), the triphones /m'ir/ and /mor/ had frequencies of 2180 and 870 ipm, respectively, in their occurrence as parts of other words. This difference was likely due to a high number of compounds and other forms derived from the high-frequency item *mir*. Note the opposite direction of the differences between the di- and triphone frequencies of the two items.

**Figure 1:**
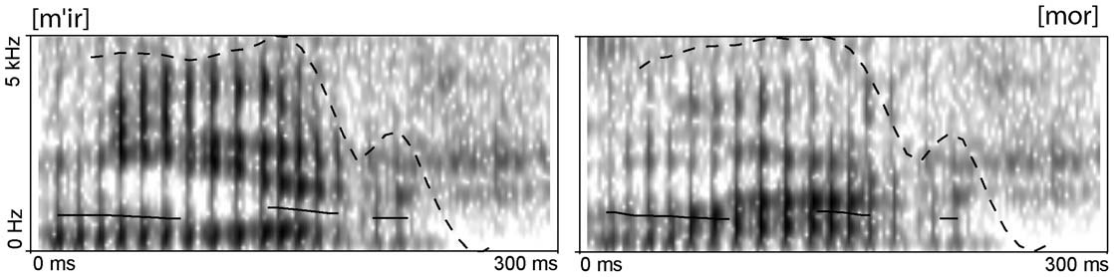
Spectrograms of the high- frequency (mir) and low-frequency (mor) words used in the experiments. The triphonemic consonant-vowel-consonant stimuli were maximally matched acoustically. Note the high similarity between the pitch (solid black line) and intensity (dashed line) contours of the two stimuli.

All stimuli (Fig. 1) were synthesized using Govorilka software package (A. Ryazanov, http://vector-ski.ru/vecs) and were matched for their duration, fundamental frequency and peak amplitude; the subjects confirmed that the stimuli were subjectively perceived as highly similar acoustcally. The stimulus length was 300 ms.

### Behavioral ratings

In a separate rating study, we assessed the psycholinguistic parameters of the stimulus words: correctness, meaningfulness, frequency, imagebility, arousal, action-relatedness, concreteness and ambiguity. To this end, ten native Russian speakers (different from EEG experiment participants) were asked to rate the stimuli on a scale of 1 to 7 using nine plain-language questions; the resulting ratings were submitted to a t-test for statistical comparison. Confirming the intended stimulus dissociation, the behavioral study participants rated *mir* (mean rating 6.3±0.6 standard deviation) as significantly (p<0.0000001) more frequent than *mor* (1.8±0.6). They also rated the high-frequency word *mir* as more ambiguous (2.8±1.4 vs. 6.4±0.6, p<0.001) and more action-related (4.5±1.4 vs. 2.2±1.3, p<0.01) than the low-frequency one. No other significant differences between the stimuli could be identified.

In addition to this independent rating study, the EEG experiment participants were asked (1) whether they considered the stimuli to be words in the Russian language and (2) whether they were familiar with these words and their meanings. All participants indicated that they viewed both the high- and low-frequency items as Russian words and were familiar with their meanings.

### Electroencephalographic recording

During the experiments, participants were seated in an acoustically shielded room and were instructed to ignore auditory stimuli and concentrate on watching a self-selected silent video. Acoustical stimuli were presented binaurally via headphones at a comfortable sound level. In each condition, a total of 667 stimuli were presented in a pseudo-random oddball sequence (85% standard and 15% deviant stimuli, with at least three standards between any two deviants). Stimulus onset-to-onset asynchrony varied randomly between 950 and 1050 ms in 10-ms steps.

During the auditory presentation, the subjects' electroencephalogram (EEG) was registered using a Telepat-104 EEG setup and 10-mm gold-plated electrodes (Potential, St. Petersburg, Russia) placed on the scalp using a reduced 10%-20% electrode configuration [30]. EEG was recorded from eleven symmetrical locations over the midline and left and right hemispheres, where the MMN can typically be found [31]: F3, Fz, F4, C3, Cz, C4, P3, Pz, P4 as well as left (LM) and right (RM) mastoid sites. The reference electrode was attached at the tip of the nose. To control for vertical and horizontal eye movements, electrooculogram (EOG) readings were taken via two electrodes placed below the left eye and lateral to its outer canthus. The sampling rate was 250 Hz. Electrode impedances were kept below 5 kOhm. On-line filtering was applied using a 0.5–70 Hz band-pass filter and a 50 Hz notch filter.

### EEG data analysis

EEG data were filtered off-line using 0.5–30 Hz band-pass filter, epoched from −40 to 600 ms relative to stimulus onset and baseline-corrected using a 40-ms pre-stimulus interval. Epochs with voltage levels exceeding ±75 µV in any EEG or EOG channel were rejected, and event-related potentials (ERPs) were then produced by separately averaging artifact-free standard and deviant trials (standard responses immediately following the deviant stimulus were discarded). Artifact-free data from all subjects except one contained at least 85 accepted deviant trials (i.e. 85% of the total number of trials presented). Data from one subject had to be excluded from the analysis due to excessive (>50%) movement-related artifacts.

The MMN response was calculated by subtracting responses to the standard stimuli from those to the deviant ones in each block. First, peak latencies of responses were obtained for each subject and condition. MMN peaks were determined as the highest amplitude of negative polarity at midline electrodes between 100 and 250 ms, when MMN peaks are most typically reported. As the analysis indicated different mean peak latencies for the two main conditions (142 vs 198 ms), we first computed average response amplitude over a large window covering both peaks (127–213 ms, i.e. starting 15 ms before the earlier peak for the high-frequency word and ending 15 ms after the later peak for the low-frequency word), and submitted this to statistical analysis. This was followed by a more refined analysis, in which 30-ms long windows defined on the basis of grand-average data for the two conditions (127–157 ms and 183–213 ms) were used for amplitude analysis. Having acquired significant results from these initial analyses, we then scrutinized the effects further and determined individual MMN peaks separately for each subject and condition. Using these, we measured ERP amplitudes by computing mean amplitude values over 30-ms windows centered on individual response maxima.

For statistical assessment of results, we performed a repeated measures analyses of variance (ANOVA) with Stimulus Type (two levels: standard vs. deviant response), Condition (high- vs. low-frequency deviant recordings), Sagittal Electrode Position (three levels: frontal, central, parietal) and Lateral Electrode Position (three levels: left, central, right) as within-subject factors followed by post-hoc tests. For direct statistical comparison of peak latencies between the MMN elicited in the two conditions, Student's t-test (two-tailed) was applied.

To further examine ERP effects and minimize acoustic confounds, we calculated identity MMN (iMMN) values by subtracting the ERPs elicited by the same sound presented as the deviant and standard stimulus in the two conditions (e.g., *mir* deviant minus *mir* standard). These were subjected to the same statistical analysis as the MMN responses, as described above.

## Results

**Figure 2:**
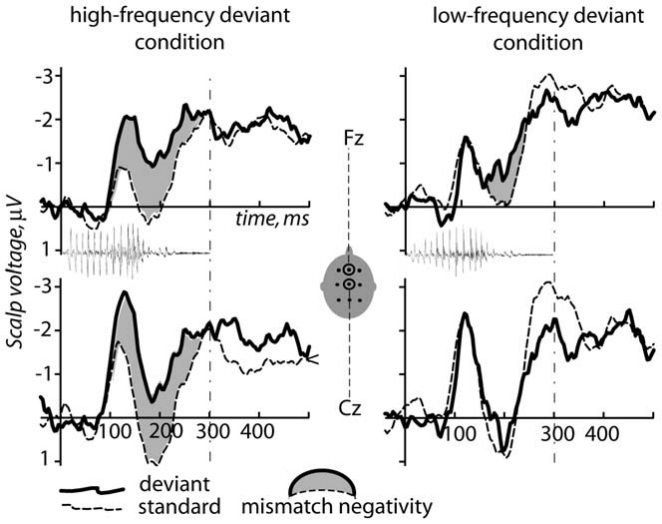
Event-related potentials elicited by standard and deviant stimuli in the high- and low-frequency deviant conditions. Midline channels with maximal ERP amplitudes are shown (superimposed on acoustic stimulus waveforms). Note the more pronounced deviant-standard difference in the high-frequency condition, whereas a smaller difference, which was also more focal (here confined to Fz), emerged in the low-frequency condition.

**Figure 3:**
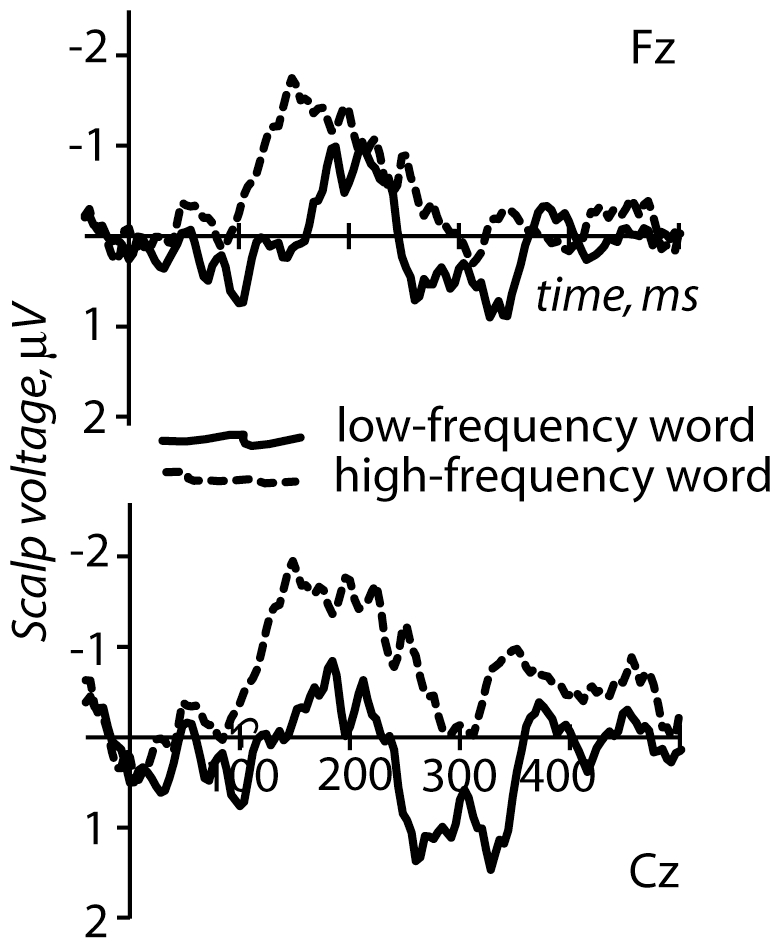
Mismatch negativity (MMN): deviant-standard difference curves for the high- and low-frequency deviant conditions. Note the more pronounced MMN for the high-frequency word and a smaller MMN for its low-frequency counterpart.

Event-related potentials were successfully calculated for the standard and deviant stimuli in both experimental conditions, and mismatch negativity responses could be obtained for both high- and low-frequency deviant words (see Figures 2 and 3).

Strong evidence of the MMN was found when the high-frequency word was used as the deviant stimulus (Fig. 2). Specifically, the mean amplitudes of standard and deviant ERPs were found to be statistically different with a significant main effect of factor Stimulus Type (i.e., deviant vs. standard; F(1,8) = 6.36, p = 0.036). In the reversed condition (with the low-frequency word as the deviant stimulus) a significant although less robust MMN was also found, as indicated by a significant difference between the deviant and standard responses (F(1,8) = 5.58, p = 0.046; Fig. 2). The main effect of the factor Sagittal Electrode Position was also significant here (F(2,16) = 6.65, p = 0.02) due to the response being more focal and restricted to the most frontal sites (while being more widespread for the high-frequency response). This interpretation was further supported by a significant interaction of Stimulus Type × Sagittal Electrode Position (F(2,16) = 7.59, p = 0.009). Post-hoc analysis indeed revealed that this interaction is due to a stronger (p<0.01) mismatch effect at more frontal sites than at centro-posterior ones.

MMN subtraction curves for both conditions are shown in Figure 3. The mean peak latency for MMN elicited by the high-frequency word was 142±14.6 ms, while that of the low-frequency word was substantially longer: 198±7.7 ms. This peak latency difference between the conditions was highly significant (Student's T(1,8) = 5.04, p<0.001).

The Condition main effect was also highly significant for the mean amplitude comparison, indicating a stronger MMN for the high-frequency deviant than the low-frequency deviant. This was true for the initial analysis covering larger window 127–213 ms (i.e. starting 15 ms before the earlier peak for the high-frequency word and ending 15 ms after the later peak for the low-frequency word), based on peaks found in grand-mean data and applied to all subjects (F(1,8) = 20.16, p = 0.003; mean Fz amplitude for *mir* −1.6 µV±0.4 µV standard error vs. *mor* −0.3±0.3 µV). This effect was also significant for the analysis of two 30-ms long time windows based around grand-average peaks (i.e. 127–157 ms and 183–213 ms; F(1,8) = 5.84, p = 0.045). Furthermore, it was highly significant when we took into account individual variability in peak latencies and computed average amplitudes of 30-ms long peaks centered on individual subjects' MMN maxima (F(1,8) = 30.63, p = 0.0009). In addition, an interaction of Condition X Lateral Electrode Position was found (F(2,16) = 6.03, p = 0.013), suggesting possible laterality differences between the conditions. Post-hoc tests, however, did not confirm significant differences between mean amplitudes at electrode positions over the left and right hemispheres.

**Figure 4:**
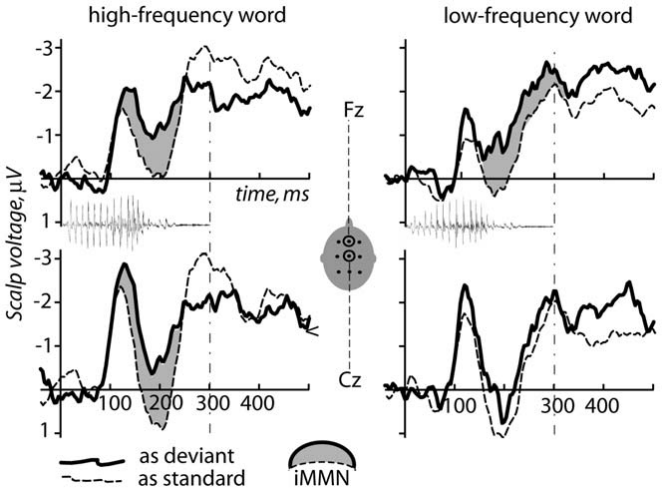
Identity mismatch negativity: direct comparison across experimental conditions between the deviant and standard responses elicited by physically identical stimuli (superimposed on acoustic stimulus waveforms). Note that the pattern of results (larger deviant-standard divergence, iMMN, for the high-frequency deviant words) is very similar to that in the original MMN analysis (cf. Fig. 2), although here the acoustic differences between the deviant and standard stimuli in each pair have been removed.

To further investigate the mismatch effects, we obtained identity MMN (iMMN) by subtracting the deviant and the standard ERPs elicited by the same sound (e.g., *mor* deviant minus *mor* standard) to rule out acoustic confounds (see Fig. 4). Significant iMMN (assessed through statistical comparison between the standard and deviant ERPs) was elicited by a high-frequency word *mir* (F(1,8) = 8.54, p = 0.019). The interaction of Stimulus type X Sagittal Electrode Position was also significant (F(2,16) = 5.65, p = 0.028), indicating a fronto-central maximum in the iMMN distribution. With respect to the low-frequency word's standard and deviant ERPs, only the Stimulus Type X Sagittal electrode position interaction proved to be significant (F(2,16) = 18.74, p<0.0001), suggesting that, even if the weak iMMN effect is present, it is focal to the most frontal (F-line) electrodes. Furthermore, when applying both a single large (127–213 ms) and two separate (127–157 ms and 183–213 ms) standardized windows based on grand-average data, we observed significantly larger iMMN amplitude for high- than low-frequency item, confirming the results obtained from conventional MMN analysis above (F(1,8) = 15.25, p = 0.0045 for two separate windows, and F = 6,52, p = 0.038 for the single wide one).

No significant main effects could be found for the factor Lateral Electrode Position in either of the two conditions.

## Discussion

In the current study, we recorded mismatch negativity responses elicited by two acoustically similar low- and high-frequency words in a passive oddball paradigm. Across the two conditions, the experimental setup and physical stimulus contrast (the critical feature for the size of acoustic MMN response) were kept identical. However, the responses elicited by these two items were markedly different. The high-frequency item elicited a stronger and earlier oddball response than the low-frequency one. Below, these results are considered in more detail:

The strongest effects on the magnitude of the MMN response are known to originate from the magnitude of acoustic contrasts between the standard and the deviant stimuli [29]: the more the stimuli diverge acoustically, the higher the MMN amplitude. The stimuli were chosen for their acoustic similarity and were carefully matched in the stimulus production process for the basic auditory features, such as fundamental frequency, peak amplitude and duration (Fig. 1). This alone means that it is not likely that the strikingly different responses can be explained by mere acoustic features. Of course, being two different words, they cannot be made fully identical acoustically. The bulk of acoustic difference between them is carried by the increased energy in lower formants for *mor* and in higher formants for *mir*, reflecting the natural phonetic distinction between /i/ and /o/. However, the standard–deviant acoustic contrast was kept identical across conditions, ruling out acoustic explanations for the observed response difference in terms of overall sound differences. Furthermore, in our analysis, we also used the ‘identity MMN’ approach, in which responses to physically identical acoustic items presented in standard and deviant positions are compared, thus further mitigating acoustic concerns. This analysis confirmed the original MMN findings and indicated a strong mismatch response to the high-frequency word with a weak iMMN effect for the low-frequency item.

Thus, as a purely acoustic explanation does not seem likely, an account based on the strong word frequency difference between the test items appears more feasible. The pattern of the difference is highly similar to that found for the word-pseudoword differences in previous studies using MMN. As reviewed in the introduction, these studies revealed higher response amplitudes for meaningful words than for meaningless, although acoustically similar, pseudowords. Pseudowords (as long as they are phonotactically legal) can also be viewed as unfamiliar words that do not normally occur in the everyday language and whose lexical frequency is therefore nil. This implies that the pattern of MMN difference between high-frequency and low-frequency words should be similar to that between a frequent word and a pseudoword ( = unknown word). This is exactly what can be concluded from comparing the current results to the previous word-pseudoword MMN studies, which is consistent with our prediction.

Previous *visual* studies of word-frequency effects on ERP amplitude suggest that the earliest differences in brain responses to the low- and high-frequency items can be found in the time range of 150–190 ms after the stimulus onset [32]. A similar time frame for lexical frequency effects, 130–190 ms, was found in other studies [33], [34], whereas slightly earlier effects (110–160 ms) were obtained when applying linear regression analyses to amplitudes of visually elicited ERPs [35]. When word frequency effects were separately studied for visual words of different length, they were found at 120–160 ms [36]. The present results, which suggest frequency effects in the time range of 140–200 ms, are thus in agreement with these previous findings, which also supports the frequency-based interpretation of the present ERP pattern. Although our findings were obtained in the auditory modality using a repetitive passive MMN design [31], they provide a good match for the visual studies mentioned above that used multiple stimulus items under attention-demanding conditions. Altogether, these studies clearly suggest early (<200 ms) neural access to lexical information regardless of the exact stimulation modality or task [2], [7].

Although word frequency is a strong candidate for explaining the current pattern of results, other psycholinguistic factors may also play a role. We analyzed these in some detail. The most obvious factor may be phonological familiarity. To assess it, we investigated di- and triphone frequencies for our test items. Diphone frequencies were of the same order of magnitude (see Methods), making it unlikely that they have introduced any bias into results. Furthermore, the diphone frequency for the initial segment /mo/ in the low-frequency word *mor* was somewhat higher than that of the other word. Because phonolological familiarity is known to lead to increased MMN responses [37], the higher diphone frequency of /mo/ should have increased the corresponding ERP. However, this was not the case, implying that the current pattern of results cannot be simply explained by differences in diphone frequencies. Triphone frequency of the complete CVC combination was higher for the high-frequency word (albeit on a different order of magnitude from diphones). This is naturally driven by the higher frequency of the word itself (as it is triphonemic) and, importantly, by the large number of semantically-related compounds and other forms derived from this noun (e.g., there are two frequent adjectives derived from *mir*, which is not the case for *mor*). Because of this, it is difficult to separate effects of word frequency for these triphonemic words from effects of triphone frequencies as such. Disentangling such sublexical and lexical frequency effects may be the task of future studies.

More generally, lexicality and familiarity are somewhat difficult to disentangle, especially in the context of spoken language. In one view, the standardized lexical frequency of a word is actually an objective measure of its familiarity in language use [38]. In addition, all lexical entries in our mental lexicon are, by definition, familiar to us, and it is therefore impossible to find a familiar language item that is not lexicalized. In previous studies, both lexicality and acoustic familiarity (for non-linguistic stimuli) have been shown to lead to enhanced event-related responses [39], [40]. In the context of the current study, however, not all facets of stimulus familiarity were reflected in the brain response. Whereas lexical frequency (and therefore a measure similar to the familiarity at the word level) was indeed reflected, the frequency of the diphones making up the words was not manifest in the MMN response. Therefore, at least the diphone frequency data are not compatible with an interpretation at the level of acoustic or phonological familiarity. More importantly, unlike those earlier studies where stimuli were either lexical/familiar or not, here familiarity was a feature of all experimental stimuli. All stimuli were familiar to the volunteers. We explicitly addressed the familiarity issue by asking the subjects to indicate whether they could identify the stimuli as words in their native language and whether they were familiar with them and knew what they meant. All of our volunteers were very familiar with both low- and high-frequency stimuli and their meanings with no differences between the stimuli; their performance in this test was at ceiling. This was further supported by the separate rating study in which no statistical differences in the correctness and meaningfulness could be found between the stimulus items. On the other hand, some psycholinguistic research has shown that the objective lexical frequency assessed by word counts in large corpora may dissociate from the ratings subjects give about the familiarity of words [41]. Taken together, these factors suggest that, while familiarity at different levels may be related to lexical frequency, the current results cannot be explained by familiarity per se. However, to better understand the relationship of familiarity effects at the acoustic, phonological and “auditory object” levels – including lexical frequency – it will be important to further investigate their brain bases in future studies using both speech and non-speech stimuli [40]. Such studies may address this issue by using non-speech control stimuli with variable occurrence frequencies, although acoustically matching these with proper linguistic items will likely be a difficult, if not impossible, task.

Other potential confounds may be related to the semantics of the individual stimuli. In a separate rating study, we compared a number of stimulus properties by asking a group of native speakers to rate the words for correctness, meaningfulness, frequency, imagebility, arousal, action-relatedness, concreteness and ambiguity. Confirming the intended stimulus dissociation, the behavioral study participants rated *mir* as significantly (p<0.0000001) more frequent than *mor*. This supports our frequency-based explanation of the ERP differences. However, the participants also found the high-frequency word *mir* more ambiguous, which may be related to its use in a number of meanings that are sometimes unrelated (e.g., in addition to the predominant meaning, *world*, it may also mean *universe*, *secular society*, *peace*, as well as the name of a well-known space station). This may imply that multiple semantic representations exist for this word-form and may become active simultaneously on its presentation, leading to an increased amplitude of the overall neural response due to summation. Although previous results have demonstrated concurrent activation of lexical neighbors [42], [43] and even increases in neurophysiological activity for ambiguous words [44], the lexical competition is also known to result in a slowdown in word responses due to inhibition (e.g. [45]). However, in this study, there was not only an enhanced but also a more rapid response observed for the more ambiguous high-frequency items. More puzzlingly, the participants also found the high-frequency word to be more action-related than the low-frequency stimulus. As neither word denotes or suggests an object that can be manipulated or an action that can be performed, our only explanation is the more frequent use of one of the words, with the closest linked action for it being articulation. No other significant differences between the stimuli could be identified in the semantic ratings. Clearly, future studies are necessary that can tackle effects of semantic features (including ambiguity) and of their interactions with lexical variables (such as frequency) on brain activity.

The increased passive oddball response for a more frequent word must have a neurobiological explanation. The distributed network approach to word representations in the brain, which we outlined in the introduction, posits that neural processing of language is subserved by strongly connected cortical neuronal ensembles [46]. The momentary ‘ignition’ of such a network entails rapid near-simultaneous activity of its subparts, which, in turn, is manifested in the overall neural response. The strength of this response therefore depends on the strength of the internal connections within each memory circuit [26], [47], [48]. When words are learned, whether in childhood or later in life, the learning process normally involves at least perception and articulation, which invariably leads to the conjunction of neural activity in a number of brain areas (sensory, motor etc.). Thus, the distribution of word-specific networks across at least temporal and inferior-frontal cortices is determined by the dual – perceptual and motor – nature of the language function, whereas the addition of other (e.g., modality-specific) cortical areas could be linked to the words' referential semantics. These networks are unified by short- and long-distance connections whose strength depends on the amount or frequency of co-activation between their subparts. The latter is due to neurobiological mechanisms of associative learning: when neurons are simultaneously active, this strengthens mutual synaptic connections between them [23], [24], [25], [49]. Thus, frequent use of a word leads to the simultaneous activation of auditory, articulatory and possibly other cortices, which results in strong neural representations comprising neurons in the co-active areas that have become linked due to synaptic mechanisms underlying associative learning [23]. Such strongly intraconnected memory circuits, when activated by the relevant input, should therefore exhibit overall stronger neural responses than those encoding a low-frequency word. This neurobiological concept can satisfactorily explain the current pattern of ERP differences found between the low- and high-frequency words.

As mentioned above, the current ERP pattern is similar to word-pseudoword differences found in the previous studies. However, there is one important difference. Whereas the previous studies reported differences in response amplitudes, here, in addition to effects in the MMN magnitude, we also find a latency difference: the mean peak latency for the high-frequency word was shorter than that for the low-frequency one (142 vs. 198 ms). This was not found in previous studies, where word and pseudoword responses exhibited largely similar latencies (see e.g. [11], [48]). Because the stimuli in both conditions diverge at the same time acoustically, pure auditory differences cannot explain the divergent latencies of mismatch response. A different explanation appears more likely: memory traces may be activated faster when they have more robust connections, and slower when their internal connectivity is weaker. In the previous studies, no memory trace activation could occur for the pseudoword, as no memory trace exists for an unknown item. The response to pseudowords may therefore predominantly reflect a neural discrimination of purely acoustic features that is in itself quite fast; little or no excitation of lexical circuits can occur under non-attend conditions [26], [47]. Here, on the contrary, a long-term memory trace *is* activated for the low-frequency item as well as for the high-frequency one. However, as the underlying neural network for the low-frequency word is not used frequently and is therefore less well integrated, its ‘ignition’ may take longer to reach its full capacity, resulting in a delay in the peak of the corresponding brain response. This corresponds well with previous behavioral data that consistently showed faster reaction times to more frequent words (e.g. [50]). In this view, the peak latency difference may provide additional support for our explanation of the result pattern as stemming from the word-frequency differences.

Whilst we place the main stress here on neurobiological interpretation of our results, such neurobiological accounts can also be viewed as mechanistic specifications of psycholinguistic models of word recognition. For instance, the speed with which the neurophysiological activations indicative of lexical processing emerge and spread throughout the brain, which happens here before the complete acoustic information about stimulus words is present in the input, is in line with the Cohort model-inspired approach to speech perception that stresses immediate and parallel access to all linguistic representations compatible with the available auditory signal [51]. Further, as the current neurophysiological activation pattern is better explained by lexical rather that phonological word properties, it may also speak to the issue of lexical vs. phonological routes of access to representations, for example, in the light of models postulating multiple routes of accessing a word representation such as the dual route processing model, which distinguishes a ‘holistic’ lexical route from a phonological one [52] (whilst dual route models were initially developed for visual words recognition, similar logic can in principle be applied in the auditory domain, see e.g. distributed cohort model [53]). In view of psycholinguistic models, future questions emerge which might also be addressable using neurophysiological methods. Looking at the precise patterns of spreading activation as specific to phonological or lexico-semantic processing, it may be possible to differentially assess such dual route models as compared with connectionist interactive activation models (such as TRACE, which would explain the lexical frequency effect as linguistic network's ability to extract and encode statistical regularities [54]), in their capacity to explain neurobiological findings (see e.g. further discussion of psycholinguistic-neurophysiological research in [2], [26]).

Finally, although we clearly favor the frequency-based interpretation of the present pattern of results in terms of both their amplitude and latency, it must also be acknowledged that the results of this first MMN study of lexical frequency effects on brain processing of unattended spoken words should be treated with due caution, as a very limited stimulus set (one token of each type) was used. Although much care was spent to optimize the stimuli, eliminate confounds and control for various factors, further research is still necessary in order to validate the present results. Future experiments that can use larger, more varied sets of words balanced for their phonological, lexical and semantic features will allow generalization of our current findings and rule out confounds related to the stimulation paradigm (cf. [55]).

### Conclusions

In sum, we found that the high-frequency stimulus led to a significantly more pronounced MMN response than the low-frequency one, a finding that is similar to earlier reports of the enhancement of word-elicited responses relative to those of meaningless pseudowords. The high-frequency item also produced an earlier response, potentially indicating more rapid access to a frequently used lexical entry. Because potential alternative explanations based on, for example, phonological or semantic factors cannot fully explain these patterns, the interpretation based on word frequency appears to be most plausible. This result supports the account of word memory traces as neuronal assemblies that can be activated automatically in an attention-free manner. The speed and magnitude of this activation may be linked to the strength of internal connections in a memory circuit, which is in turn determined by the everyday use of language elements. Further research is necessary to fully explore interactions between acoustic, phonological, lexical and semantic variables in early neural processing of spoken words.
